# Real-world management of heart failure in the Netherlands: Improving quality of care by simple measures

**DOI:** 10.1007/s12471-018-1110-8

**Published:** 2018-04-10

**Authors:** S. Koudstaal, F. W. Asselbergs

**Affiliations:** 1 Department of Cardiology, University Medical Centre Utrecht, Utrecht, The Netherlands; 2 Farr Institute of Health Informatics Research, London, UK

In the era of evidence-based medicine, clinicians experience on a daily basis that its holy grail, the randomised clinical trial, excels at internal validity but frequently lacks external validity. In fact, only 13% of patients seen with heart failure in daily clinical practice would have matched the stringent inclusion and exclusion criteria in heart failure clinical trials [1]. As a result, everyday discrepancies are still largely resolved by the clinical judgement of the treating physician. On the other hand, we need trials to test safety and efficacy of new drugs. In the field of heart failure, we have seen the introduction of first-in-class drugs that harness the potential to bring about substantial improvements in heart failure care and survival [2]. Hence, guidelines for heart failure treatment are frequently and thoroughly updated, in particular for heart failure with reduced ejection fraction (HFrEF) [3, 4]. Collectively, there is a need to connect these two worlds to promote the uptake and implementation of evidence-based medicine in the real world. There is a growing recognition that real-world registries can increase our awareness of the huge gap between daily clinical practice and clinical trials [5–9]. So far, there have been heart failure registries that assessed medication use, but their demographics still showed there is considerable residual selection bias as these registries still lack patients who frequently are underrepresented in clinical trials, such as women, the elderly, and patients with multiple comorbidities [10].

In this issue of the Netherlands Heart Journal, Brugts et al. present the framework and forthcoming of a new real-world registry in the Netherlands that included over 10,000 patients with heart failure, called CHECK-HF [11]. Unselected patients diagnosed with chronic heart failure at Dutch outpatient clinics were included, of those the vast majority being diagnosed with HFrEF (79%). With a mean age of 73 years and 40% of patients being female, CHECK-HF proves to be a better resemblance of heart failure seen in the real world than previous heart failure registries. Medication uptake was carefully recorded, including dosages of drugs. Particularly the latter will be helpful in understanding how well we are treating heart failure. The central question is, can we improve guideline adherence by simply measuring?

It comes as no surprise that quality of care can be considerably improved by simply making best use of the therapeutics we already have. For example, it is known for more than a decade that black Americans are among those with the highest hypertension-related mortality and that interventions with calcium channel blockers and angiotensin converting enzyme inhibition in these patients are most effective [12]. Yet, only very recently, the New England Journal of Medicine published a cluster-randomised intervention that aimed to measure and intervene on high blood pressure levels at the patients’ local barbershop. This simple intervention led to a substantial decline in uncontrolled hypertension [13]. Novel intervention, old drugs. Why should it be any different in the field of heart failure? The authors believe that heart failure prescription rates in the real world are modest at best and that renin angiotensin system antagonists and/or betablockers dosages are only sporadically on target levels. If that is confirmed in a contemporary cohort such as CHECK-HF, then the real contribution to improving heart failure care is increasing the guideline adherence and use what’s already out there. No doubt that if all HFrEF patients are treated with adequate dosages of neprilysin inhibitors/angiotensin II receptor blockers, betablockers, mineralocorticoid receptor antagonists, receive iron supplementation when iron deficient, are actively counselled with regard to lifestyle habits, and, last but not least, receive appropriate device therapy, future heart failure trials will probably look increasingly similar to clinical trials in the field of acute coronary syndromes and antithrombotic agents—that is, we will need to enrol more than 15,000 patients to detect minute differences in mortality on a statistical level.
